# Eating on nightshift: A big vs small snack impairs glucose response to breakfast

**DOI:** 10.1016/j.nbscr.2017.12.001

**Published:** 2017-12-07

**Authors:** Stephanie Centofanti, Jillian Dorrian, Cassie Hilditch, Crystal Grant, Alison Coates, Siobhan Banks

**Affiliations:** a Sleep and Chronobiology Laboratory, University of South Australia, Adelaide, South Australia, Australia; b Alliance for Research in Exercise, Nutrition and Activity, University of South Australia, Adelaide, South Australia, Australia

**Keywords:** Simulated shift work, Glucose response, Metabolism, Diet, Diabetes, Nightshift

## Abstract

Shift work is a risk factor for chronic diseases such as Type 2 diabetes. Food choice may play a role, however simply eating at night when the body is primed for sleep may have implications for health. This study examined the impact of consuming a big versus small snack at night on glucose metabolism. N = 31 healthy subjects (21–35 y; 18 F) participated in a simulated nightshift laboratory study that included one baseline night of sleep (22:00 h-07:00 h) and one night awake with allocation to either a big snack (2100 kJ) or small snack (840 kJ) group. The snack was consumed between 00:00–00:30 h and consisted of low fat milk, a sandwich, chips and fruit (big snack) or half sandwich and fruit (small snack). Subjects ate an identical mixed meal breakfast (2100 kJ) at 08:30 h after one full night of sleep and a simulated nightshift. Interstitial glucose was measured continuously during the entire study using Medtronic Continual Glucose Monitors. Only subjects with identical breakfast consumption and complete datasets were analysed (N = 20). Glucose data were averaged into 5-minute bins and area under the curve (AUC) was calculated for 90 min post-breakfast. Pre-breakfast, glucose levels were not significantly different between Day1 and Day2, nor were they different between snack groups (p > 0.05). A snack group by day interaction effect was found (F_1,16_ = 5.36, p = 0.034) and post-hocs revealed that in the big snack group, AUC response to breakfast was significantly higher following nightshift (Day2) compared to Day1 (p = 0.001). This translated to a 20.8% (SEM 5.6) increase. AUC was not significantly different between days in the small snack group. Consuming a big snack at 00:00 h impaired the glucose response to breakfast at 08:30 h, compared to a smaller snack. Further research in this area will inform dietary advice for shift workers, which could include recommendations on how much to eat as well as content.

## Highlights

• Eating at night when the body is primed for sleep may have implications for health of shift workers.
• This study examined the impact of consuming a big versus small snack at night on glucose metabolism.
• Consuming a big snack at midnight impaired the glucose response to breakfast at 08:30 h, compared to consuming a smaller snack at midnight.

## 1 Introduction

Some 20% of the population are required to work outside the regular 09:00–17:00 h working day, and this number is likely to increase as economic demands push work hours into the night for many industries (Rajaratnam and Arendt, 2001). These irregular schedules mean workers often have to sleep during the day and be awake at night. This causes a misalignment between normal day-light entrained internal physiological processes, such as metabolism and digestion, and the external environment (Van Cauter et al., 1991, Banks and Dinges, 2007). As a consequence, shift workers have poorer health than day workers, even after controlling for lifestyle and socioeconomic status, with increased risk of obesity and Type 2 diabetes (Banks et al., 2014).

Night shift workers tend to redistribute meals from the day to night hours (Banks et al., 2014). Humans are biologically primed to eat during the daytime, with lower hunger ratings (Heath et al., 2012), slower gastric emptying (Goo et al., 1987), reduced glucose tolerance (Van Cauter et al., 1992), increased insulin resistance (Morgan et al., 1999) and impaired insulin secreting β-cell function (Rakshit et al., 2015) during night-time hours. Eating during night hours may therefore have negative consequences for metabolism. Indeed, studies have shown that eating late in the day reduces the effectiveness of weight loss programs independent of energy intake, dietary composition or sleep duration (Garaulet et al., 2013), and that meals consumed after 20:00 h predicted higher body mass index (BMI) even after controlling for sleep timing and duration (Baron et al., 2011).

Preliminary laboratory work by our research group has compared glucose response to a standard breakfast meal across four simulated nightshifts in a group of young healthy males (Grant et al., 2017). Participants were randomised to two conditions. In one condition participants received a large meal in the middle of the night (01:00 h), and in the other, food intake was redistributed to daytime hours, keeping total 24-hour energy intake constant. In the group who ate at night, glucose area under the curve (AUC) significantly increased across the nightshifts, but remained relatively stable for those who only ate during the day. These results suggest that refraining from eating during the night may limit impairments to glucose metabolism (Grant et al., 2017). Therefore, recommending that night-workers avoid eating during their shifts may reduce risk of metabolic disturbance for this group. A clear limitation to this approach is the potential that workers will not tolerate complete redistribution of food intake to outside work hours. However, it may be possible to limit changes in glucose metabolism by simply reducing, rather than eliminating food at night. Therefore, the aim of this study was to examine the impact of consuming a big versus small snack during a simulated night shift on glucose response to a standard breakfast meal the next morning.

## 2 Materials and methods

### 2.1 Subjects

Thirty-two healthy adult volunteers were recruited. One subject withdrew due to illness part-way through the study. The mean age (±SD) of the remaining 31 subjects was 24.3 (± 3.4) years (range: 21–35 years; 18 female). The average body mass index (BMI) was 22.2 ± 3.0 kg/m^2^; participants with a BMI of over 25 kg/m^2^ were excluded from participation. To meet inclusion criteria subjects were required to sleep a minimum of 7 h per night with bedtime no later than midnight and wake time before 09:00 h for the week prior to the laboratory phase of the study. This routine was confirmed using sleep diaries, wrist actigraphy and time-stamped messages each morning. During this period, subjects were not allowed to nap, consume caffeine nor alcohol.

Subjects were excluded from the study if they reported: being a smoker; drinking more than two cups of caffeinated drinks or two standard drinks of alcohol per day; trans meridian travel in the past three months; shift work in the past two years; a BMI above 25 kg/m^2^; current medication (apart from the contraceptive pill) or recreational drug use (illicit drugs confirmed by urine test); and any medical, psychological or sleep disorders. Blood chemistry analysis was conducted to confirm general health. The study was approved by the University of South Australia Human Research Ethics Committee. Subjects gave written, informed consent and were reimbursed for their time.

### 2.2 Protocol

Subjects resided in a windowless and sound-attenuated sleep laboratory. Ambient room temperature was maintained at 22 ±1 °C. Light intensity was set to <50 lx at head height (dim light) during all wake periods of the protocol, and <0.03 lx (darkness) during all scheduled sleep periods.

**Fig. 1:**
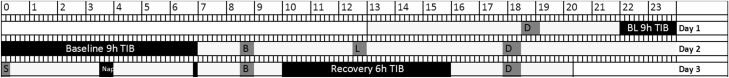
Schematic represents study protocol. Time of day is presented across the X-axis, from midnight to midnight on Study Days 1, 2, and 3. Black boxes represent time in bed opportunities (TIB). The TIB opportunity ending at 04:00 h represents the 30-minute and 10-minute night time nap for each nap condition respectively (30-NAP; 10-10-NAP). The TIB opportunity ending at 07:00 h represents the 10-minute morning nap opportunity for the 10-10-NAP condition. Meals are shown in the grey boxes (B= breakfast, L=lunch, D=dinner, S=Snack). Across both snack conditions, each breakfast comprised of ~2100 kJ, lunch comprised of ~2700 kJ and each dinner comprised of ~3400 kJ. The big snack condition comprised of ~2100 kJ and the small snack condition comprised of ~840 kJ.

Subjects spent two nights and three days in the sleep laboratory: one day for adaptation and training, one baseline day and night, one experimental night of sleep deprivation, and one recovery day. Subjects arrived at the laboratory at 13:00 h and spent the adaptation day practicing various performance tasks. They had a 9-hour sleep opportunity from 22:00 h to 07:00 h on the first night (Fig. 1).

On the second night, subjects participated in a simulated night shift. All subjects had an identical dinner meal at 18:00 h (~3400 kJ). Subjects were then randomly allocated to either a big snack (~2100 kJ, *n* = 15) or small snack (~840 kJ, *n* = 5) group. The snack was consumed between 00:00–00:30 h and consisted of low fat milk, a sandwich, chips and fruit (big snack) or half sandwich and fruit (small snack). The macronutrient composition of the snacks is presented in Table 1. Subjects ate an identical mixed meal breakfast (2100Kj; Table 1) at 08:30 h after a full night of sleep (Day1) and a simulated nightshift (Day2). All food was prepared and monitored by qualified research staff, and subjects were not permitted to eat outside of set meal times. Interstitial glucose was measured continuously during the entire study using Medtronic Continual Glucose Monitors, with sensors placed in the subcutaneous layer of the participants’ medial abdominal area. Glucose levels were extracted in mg/dL using Medtronic CareLink Pro 3.3 software (Medtronic MiniMed) and converted to mmol/L by dividing values by 18.

**Table 1:** Macronutrient composition for the test meal (breakfast), the big snack and small snack.

	**Foods (quantity)**	**Energy (kJ)**	**Total fat (g)**	**Protein (g)**	**Carbohydrate (g)**	**Fibre (g)**
**Breakfast**						
	Orange juice (200 mL)	245	0.0	1.5	12	0.4
	Skim milk (200 mL)	297	0.2	7.5	10	0.0
	Cereal (53 g)	822	0.3	4.6	42.5	1.6
	Yoghurt (200 g)	778	2.0	10.0	30.6	0.0
	**Total**	**2142**	**2.5**	**23.6**	**95.1**	**2.0**
**Small snack**						
	White bread (1slice)	278	0.5	2.7	12.3	0.8
	Peanut butter (0.5 tbsp)	285	4.8	2.1	3.4	1.3
	Orange (medium size)	281	0.1	1.7	12.4	4.3
	**Total**	**844**	**5.4**	**6.5**	**28.1**	**6.4**
**Big snack**						
	White bread (2 slices)	556	1.0	5.4	24.5	1.6
	Peanut butter (1 tbsp)	570	9.6	4.3	7.5	2.7
	Orange (medium size)	281	0.1	1.7	12.4	4.3
	Skim milk (200 mL)	295	0.2	7.5	10.0	0.0
	Potato chips (19 g)	410	6.4	1.1	8.7	0.7
	**Total**	**2112**	**17.3**	**20.0**	**63.1**	**9.3**

Note: g, grams; kJ, kilojoules; mL, millilitres; tbsp, tablespoon.

As part of a larger study (Centofanti et al., 2016, Centofanti et al., 2015, Hilditch et al., 2015, Hilditch et al., 2015), participants were assigned to one of three groups: a control group (NO-NAP, *n* = 6); a 10 min “on-shift” nap ending at 04:00 h plus a 10 min “top-up” nap at 07:00 h (10-10-NAP, *n* = 5, mean combined sleep time=16.6 min, SE=0.9); or a 30 min “on-shift” nap ending at 04:00 h (30-NAP, *n* = 9, mean sleep time=26.3 min, SE=0.9).

On the final day of the study, subjects were allowed a 6-hour daytime recovery sleep opportunity between 10:00 h and 16:00 h. Sleep was measured using polysomnography (PSG) during all sleep periods with the Compumedics Grael Sleep System and Compumedics Profusion PSG 3 Software (Melbourne, Australia). Placement of electrodes was in line with the 10/20 system of electrode placement (Carskadon and Rechtschaffen, 1994). Sleep data were scored in 30-second epochs in accordance with the criteria of Rechtschaffen and Kales (1968) and total sleep time (TST) was derived.

During wake periods, subjects performed neurobehavioral test batteries approximately every 2 h and were permitted to read books, play card/board games, watch DVDs, interact with each other and study staff, or listen to music between test sessions. Subjects did not have access to any clock-bearing or telecommunication devices. Subjects were not allowed to perform any vigorous activities during the study.

### 2.3 Statistical analysis

Analyses were performed using SPSS Statistics Version 21.0 (IBM Corp., Armonk, NY, USA). Only subjects with identical breakfast consumption and complete glucose datasets were analysed (*n* = 20). Glucose data were averaged into 5-minute bins and area under the curve (AUC) was calculated for 90 min post-breakfast, with baseline (BL) glucose values calculated as the average of the three pre-breakfast points: 08:15 h, 08:20 h, and 08:25 h. AUC was computed using the trapezoidal estimation method (Venn and Green, 2007) to demonstrate overall glucose response to breakfast. A linear mixed model ANOVA (Van Dongen et al., 2004) was conducted to assess the effects of snack group (big snack [NO-NAP *N* = 4, 10-10-NAP *N* = 4, 30-NAP *N* = 7]; small snack [NO-NAP *N* = 2, 10-10-NAP *N* = 1, 30-NAP *N* = 3]), and day (Day1=following one full night of sleep; Day2=following simulated night shift), and the snack group*day interaction on glucose AUC response to breakfast. As this was part of a larger napping study which involved allocation to a short night-time nap (NO-NAP, 10-10-NAP, 30-NAP), the model also specified predictors of nap group and a nap group*day interaction term to account for any potential effects of the short naps between groups on glucose response to breakfast.

**Fig. 2:**
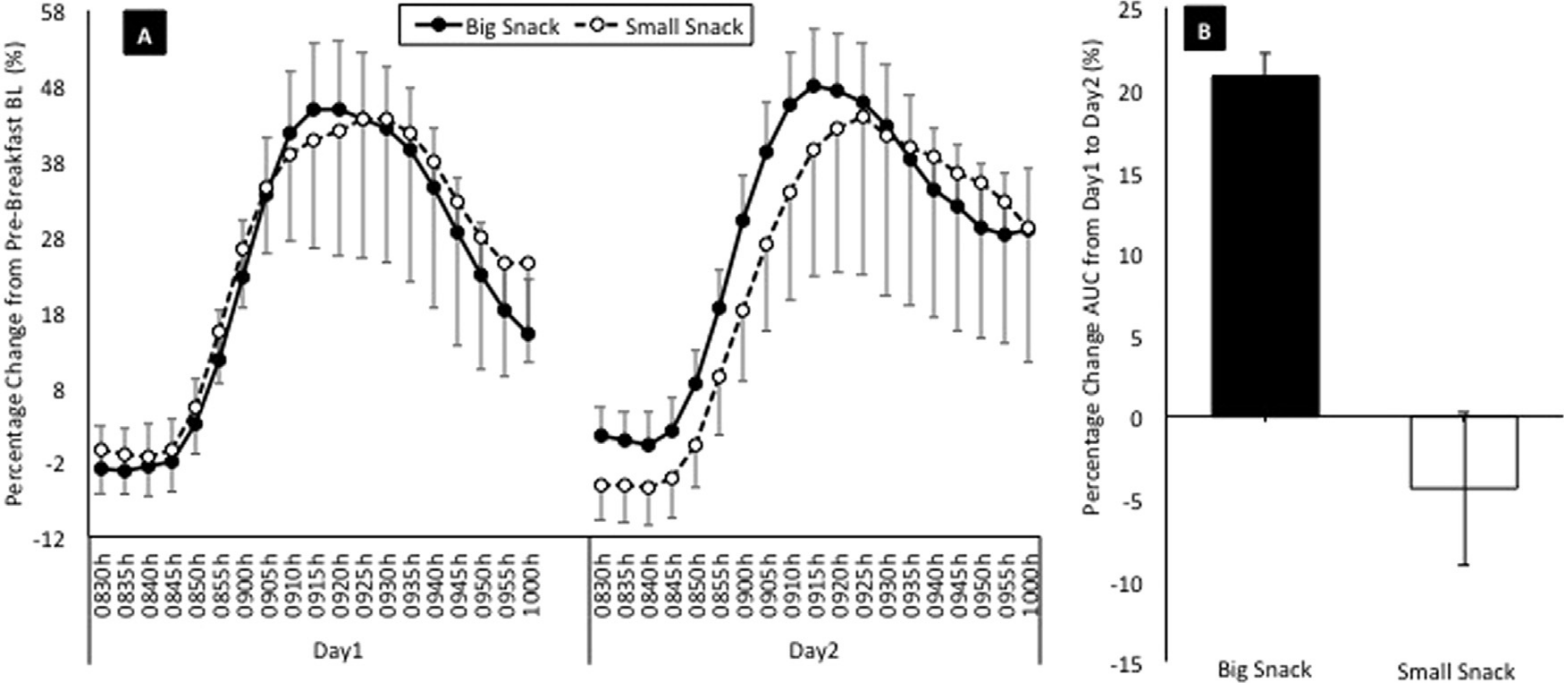
**Panel A**: Mean glucose data in 5-minute bins for 90 min post-breakfast on Day1 (following a night of sleep) and Day2 (following one simulated night shift) expressed as a percentage change from baseline (BL=average of three pre-breakfast points: 08:15 h, 08:20 h, and 08:25 h, on each day). Open circles represent the small snack group (840 kJ at 00:00 h). Closed circles represent the big snack group (2100 kJ at 00:00 h). Bars represent standard error of the mean. **Panel B:** Percentage change Area Under the Curve (AUC) from Day1 to Day2 in the big (black column) and small (white column) snack groups. Bars represent standard error of the mean.

Mean pre-breakfast BL glucose values for each group were: big snack=4.0 mmol/L (SEM=0.2); small snack=4.6 mmol/L (SEM=0.3); NO-NAP=4.8 mmol/L (SEM=0.3); 10-10-NAP=3.9 mmol/L (SEM=0.2); and 30-NAP=3.9 mmol/L (SEM=0.4). To account for baseline differences in analyses, linear mixed models specified a random effect of subject ID, allowing different intercept values for each participant. Data in Fig. 2 are presented as change relative to BL (average of 3 pre-breakfast timepoints) and relative to Day1 AUC for ease of interpretation.

## 3 Results

On average, mean glucose response to breakfast was within a normal, healthy range (3–7 mmol/L 2 h post-glucose challenge is defined as healthy [American Diabetes Association, 2014]) across both study days. Mean glucose levels in the 90-minutes post-breakfast on Day1 and Day2 are presented for each snack condition and nap condition in Table 2.

**Table 2:** Mean glucose levels in the 90-minutes post-breakfast on Day1 and Day2 for each snack condition and nap condition.

	*N*	Day1 M(SD) mmol/L	Day2 M(SD) mmol/L
**Snack groups**			
Big snack	15	4.9 (0.2)	5.9 (0.3)
Small snack	5	5.8 (0.5)	5.6 (0.6)
**Nap groups**			
NO-NAP	6	6.0 (0.5)	6.1 (0.5)
10-10-NAP	5	4.8 (0.5)	6.2 (0.6)
30-NAP	9	4.7 (0.7)	5.4 (0.3)

Notes: M=mean; N=number; SD=standard deviation

There were no significant main effects of snack group (*F*_1,16_ = 0.14, *p* = 0.713), nap group (*F*_1,16_ = 2.03, *p* = 0.164) or day (*F*_1,16_ = 3.06, *p* = 0.99), nor was there a significant nap group*day interaction (*F*_1,16_ = 1.93, *p* = 0.177). However, a snack group*day interaction was found (*F*_1,16_ = 5.36, *p* = 0.034), such that following one night awake, glucose AUC response to breakfast was significantly greater than on Day1 in the big (p = 0.001), but not the small (p > 0.05) snack group (Fig. 2). This translated to a 20.8% (SEM 5.6) increase in AUC from Day1 to Day2 for the big snack group.

## 4 Discussion

This study found that the glucose response to a standard breakfast, following one night awake, was impaired in subjects who ate a big snack (2100 kJ) compared to those who ate a small snack (840 kJ) at midnight.

These results are in line with other studies showing reduced glucose tolerance following night-time meals, despite overall energy intake remaining constant (Grant et al., 2017, Bonham et al., 2016). Findings from previous studies suggest that not eating during night shifts may reduce the risk of metabolic disturbance for night-workers (Grant et al., 2017, Bonham et al., 2016). However, complete redistribution of food to outside work hours may not be tolerated by all workers. To our knowledge, the current study is the first to show that simply reducing the amount of food consumed at night, rather than eliminating food intake at night completely, may limit impairments in glucose metabolism in humans. These results also suggest that eating a big snack at night impacts glucose metabolism over and above a night of sleep deprivation alone (Morris et al., 2015).

In the current study, having a 30-minute nap or two 10-minute naps during a simulated night shift did not have a significant effect on the glucose response to breakfast. It should be noted, however, that this study was not designed to test the effects of the naps on glucose tolerance. Due to the limited sample size in the current study, it would be beneficial to investigate the potential impact of naps in a larger sample. Moreover, the nap duration was short, with total sleep time of less than 30 min. Given the relationship between glucose metabolism and sleep loss shown in previous studies (e.g. Reynolds et al., 2012), it is possible that longer naps (e.g. >60 min) may improve glucose metabolism; future studies should investigate this further. However the design of the current study, which involved a full night of sleep deprivation and a short nighttime nap, replicates a schedule which is common in rotating shift workers, who are often unable to prophylactically nap prior to the first in a series of night shifts and thus experience >24 h of sleep deprivation.

As well as changing the timing of food intake, shift workers have also been observed to have different macronutrient compositions compared to day workers. For example, studies have found that shift workers have a higher intake of carbohydrates and saturated fat than day workers (Schiavo‐Cardozo et al., 2013, Morikawa et al., 2008). Future investigations into the ideal macronutrient composition of snacks eaten at night are required. The snacks in the current study were comprised of carbohydrate-rich foods. It would be helpful to replicate this study with lower carbohydrate foods, which may help to reduce the glucose spike following a subsequent meal (Higgins, 2012). In addition, although energy intake was maintained between subjects up until the time of the snack, the big snack group had an excess of kilojoules compared to the small snack group because of the different energy levels of the snacks. This positive energy balance may be contributing to the reported findings of an impaired glucose response to breakfast in the big snack condition. However, converging evidence from studies assessing different night shift meal sizes has shown that eating more during the night worsens the glucose response to breakfast even when energy intake is distributed evenly between conditions over 24 h (Grant et al., 2017).

Gastric emptying rate, which can affect the glucose response to a subsequent meal (Marathe et al., 2015), should also be taken into account, especially as previous studies have reported lower gastric emptying rate at night (Grammaticos et al., 2015) which may be further impacted with circadian misalignment (Konturek et al., 2011). Given the difference in macronutrient content and size of the snacks, it is possible that the rate of gastric emptying in the 8-hours between the snack and breakfast may have differed between the big and small snack groups and affected post-prandial glucose response (Ribeiro et al., 1998). However, given the length of time between the snack and breakfast, it is also likely that any post-prandial effects would have already subsided by 08:30 h. It is also unlikely due to the short duration of the laboratory protocol would have caused circadian disruption that may have impacted glucose results. Participants were healthy individuals with a regular sleep schedule. Thus having one night of sleep deprivation where the sleep wake schedule was mismatched to their circadian rhythms is unlikely to have resulted in shifted circadian phase. Future studies should investigate the effects of circadian misalignment on glucose response further given that chronic circadian misalignment is common in shift workers.

Glucose levels normally rise and then return to baseline within a few hours of consuming a meal (Röder et al., 2016). However, if glucose levels are chronically elevated or exaggerated after a standard meal, there may be long-term implications including increased risk of Type 2 diabetes and heart disease (Giugliano et al., 2008). The current study showed an acute impairment to glucose metabolism after consuming a large night-time snack, in a controlled environment, with a small number of healthy, lean adults. Despite impairments in the glucose response to breakfast in the big snack group on Day2, the average glucose response remained within the healthy range across both study days (~3–7 mmol/L). Shift workers have high rates of obesity (Suwazono et al., 2008) and are chronically sleep deprived (Dinges et al., 2005), two factors that contribute to metabolic disruption. Therefore, in order to more accurately assess the effects of reducing food intake at night on metabolic impairment in shift workers, further studies are needed in larger samples comprising shift working populations, who are chronically sleep deprived and have high rates of comorbid illnesses.

It should be noted that there were differences in glucose response to breakfast between the groups at baseline. However, since these baseline responses were well within normal range (Caumo, 2000), the chance that they were associated with exaggerated or blunted responses to breakfast following a night awake is negligible. In addition, this study assessed glucose response to a breakfast meal in order to simulate a naturalistic metabolic challenge. Future studies should incorporate measures of plasma glucose, insulin, and lipids in response to oral or intravenous glucose tolerance tests. This would allow for a more rigorous examination of the mechanisms underlying the glucose impairments observed after eating large amounts at night, especially given known differences between plasma glucose and interstitial glucose measures whereby interstitial glucose levels lag behind plasma glucose levels (Kulcu et al., 2003). Participants in the current study undertook a daytime recovery sleep from 10:00 h (90-minutes post-breakfast). Analyses in future studies should be extended beyond 90-minutes to observe longer-term glucose responses to a test meal and assess how long glucose levels take to return to baseline levels.

Based on these preliminary findings, it appears that eating a small snack on night shift does not impair glucose metabolism in the short term. Eating a small snack may be a beneficial recommendation to reduce the high risk of metabolic disorders observed in shift workers, especially for night-workers who find it difficult to abstain from eating altogether on-shift. Shift workers also have increased gastrointestinal complaints compared to day workers (Knutsson and Bøggild, 2010). Small night-time meals may be a strategy to reduce gastrointestinal complaints; future studies are needed which take gastrointestinal symptoms into account when investigating the effects on night-time meal size on glucose metabolism. As well as physical impacts, recent laboratory work suggests that eating a large meal at night may also have a negative impact on cognitive performance compared to abstaining from food during a simulated night shift, as measured by a psychomotor vigilance task and driving simulator test (Gupta et al., 2017). Therefore, future investigations into meal size during the night should also measure cognitive performance in order to make optimal recommendations for health and safety.

In conclusion, consuming a big snack at midnight impaired the glucose response to breakfast, relative to a smaller snack. Further research in this area will inform dietary advice for shift workers. Recommendations on how much to eat as well as meal quality and timing are needed to assist with reducing the high rates of metabolic disturbance seen in shift working populations.
